# Analysis of the Serotonergic System in a Mouse Model of Rett Syndrome Reveals Unusual Upregulation of Serotonin Receptor 5b

**DOI:** 10.3389/fnmol.2017.00061

**Published:** 2017-03-08

**Authors:** Steffen Vogelgesang, Sabine Niebert, Ute Renner, Wiebke Möbius, Swen Hülsmann, Till Manzke, Marcus Niebert

**Affiliations:** ^1^DFG Research Center and Excellence Cluster Microscopy at the Nanometer Range and Molecular Physiology of the BrainGöttingen, Germany; ^2^Department of Maxillofacial Surgery, University Medical CenterGöttingen, Germany; ^3^Department of Neurogenetics, Max Planck Institute of Experimental MedicineGöttingen, Germany; ^4^Clinic for Anesthesiology, University Medical CenterGöttingen, Germany; ^5^Institute of Neuro- and Sensory Physiology, University Medical CenterGöttingen, Germany

**Keywords:** mouse models, 5-HT receptors, 5-ht5b receptor, Rett syndrome (RTT), MeCP2, serotonin

## Abstract

Mutations in the transcription factor methyl-CpG-binding-protein 2 (MeCP2) cause a delayed-onset neurodevelopmental disorder known as Rett syndrome (RTT). Although alteration in serotonin levels have been reported in RTT patients, the molecular mechanisms underlying these defects are not well understood. Therefore, we chose to investigate the serotonergic system in hippocampus and brainstem of male *Mecp2^-/y^* knock-out mice in the B6.129P2(C)-Mecp2(tm1.1Bird) mouse model of RTT. The serotonergic system in mouse is comprised of 16 genes, whose mRNA expression profile was analyzed by quantitative RT-PCR. *Mecp2^-/y^* mice are an established animal model for RTT displaying most of the cognitive and physical impairments of human patients and the selected areas receive significant modulation through serotonin. Using anatomically and functional characterized areas, we found region-specific differential expression between wild type and *Mecp2^-/y^* mice at post-natal day 40. In brainstem, we found five genes to be dysregulated, while in hippocampus, two genes were dysregulated. The one gene dysregulated in both brain regions was dopamine decarboxylase, but of special interest is the serotonin receptor 5b (5-ht_5b_), which showed 75-fold dysregulation in brainstem of *Mecp2^-/y^* mice. This dysregulation was not due to upregulation, but due to failure of down-regulation in *Mecp2^-/y^* mice during development. Detailed analysis of 5-ht_5b_ revealed a receptor that localizes to endosomes and interacts with G_αi_ proteins.

## Introduction

Rett syndrome (RTT) is a progressive neurodevelopmental disorder caused by different mutations in the X-chromosome-linked MECP2 gene (Amir et al., 1999). With an estimated prevalence of 1 in 10,000 live births (OMIM 312750, Rett, 1966), RTT is the second most common cause for mental retardation in females after Down syndrome (Percy and Lane, 2004; Fehr et al., 2011) and usually results from maternal or paternal *de novo* germline mutations (Thomas, 1996; Girard et al., 2001), but is rarely transmitted through generations (Hoffbuhr et al., 2001). Affected children initially develop apparently normal, but after 6–18 months, rapid developmental regression occurs where they lose previously learned skills. Regression eventually halts, but is accompanied by accelerated motor deterioration (Gillberg, 1986; Zappella, 1997). Characteristic features of RTT can be divided into mental disabilities like cognitive impairment, loss of speech, anxiety and seizures and bodily ailments. The latter can be grouped into central dysfunctions like apraxia, ataxia and dystonia or disturbed respiration and into autonomous dysfunctions like constipation, oropharyngeal dysfunctions, cardiac abnormalities, and osteopenia (Mount et al., 2001; Mari et al., 2005).

There are many different mouse models available that resemble classical RTT (Ricceri et al., 2008). Although only 10% of all human mutations create a true null phenotype (Katz et al., 2012), we chose the B6.129P2(C)-Mecp2(tm1.1Bird) mouse model which shows a complete deletion of exons 3–4 (Guy et al., 2001). A detailed comparison of all available models found little differences between mouse lines regarding principle changes of morphology, autonomic or motoric functions and lifespan or neurological features, while the major differences were found in cognition and behavior (Katz et al., 2012). However, substantial differences in the time of onset and degree of severity between different models have been reported (Chen et al., 2001; Guy et al., 2001; Shahbazian et al., 2002).

The serotonergic system is one of the oldest neuro-modulatory networks, and serotonergic dysfunction has been linked to similar symptoms as listed above in other genetic disorders including Prader–Willi or sudden infant death syndrome (Saito et al., 1999; Weese-Mayer et al., 2003; Waters, 2010). Regarding RTT, some groups reported reduced levels of serotonin in different brain regions and in the cerebrospinal fluid both in human patients as well as in Mecp2^-/*y*^ mice (Jellinger, 2003; Ramaekers et al., 2003). Projecting fibers from the raphé nuclei innervate basically every brain region in mammals, although no alterations in the raphé nuclei and its ascendant serotonergic fibers were observed in Mecp2^-/*y*^ mice at P21 and P56 (Santos et al., 2010). However, until now there is no information about specific genes involved in the serotonergic transmission in *Mecp2^-/y^* mice. With no apparent alterations in the area of origin, we decided to compare the regulation of the entire serotonergic system in two distinct brain areas between healthy wild type mice and Mecp2^-/*y*^ littermates that receive considerable serotonergic projections. Here, a single transmitter activates 13 different molecular targets, 12 being G-protein-coupled receptors (GPCRs) and one (5-HT_3_R) is an ion channel. Adding to the count of 13 serotonergic targets, 3 enzymes are associated with serotonin production or transport. While RTT and related syndromes are so far incurable, progress has been made with pharmacological treatment, targeted at multiple GPCR populations in general (Wang et al., 2015) and at the serotonergic system specifically. For example, the application of 5-HT_1A_ agonists was described to have beneficial effects on the respiratory phenotype in mice (Abdala et al., 2010), while pharmacological treatment with 5-HT_7_ agonists was reported to improve behavior (De Filippis et al., 2014, 2015).

Considering the delayed onset of symptoms in RTT, we chose to compare gene expression levels at P40 in male *Mecp2^-/y^* knockout mice. Males at P40 displayed sufficiently developed, uniform symptoms and were still healthy enough to collect sufficient numbers for analysis. For this study we reasoned that the complete lack of MeCP2 will better reveal any regulatory effect than a highly variable *Mecp2* expression in hemizygous female mosaics.

## Materials and Methods

### Ethics Statement

The experimental procedures were performed in accordance with European Community (EU Directive 2010/63/EU for animal experiments) and National Institutes of Health guidelines for the care and use of laboratory animals. The study was approved by the Georg-August-University, Göttingen and the approval ID T12/18 was assigned to this work.

### Nomenclature

Following with convention, we designated human genes with all capital letters in italics (e.g., *MeCP2*), murine genes are given in italics with a starting capital letter (e.g., *Mecp2*) and genotypes are designated by superscript letters with “+” designating the presence of the wild type allele, “-” designating the knock-out of the allele and “y” indicating the male chromosome. Proteins are given in capital letters not differentiating between human and murine origin (e.g., MeCP2). For serotonin receptors, this takes the form of 5-HT_x_R, with x designating the subtype. The exception here are the members of serotonin receptor family 5, whose members are given in the form 5-ht_x_R.

### Animals

The *Mecp2* knockout mouse (*Mecp2^-/y^*), which is a model for RTT, strain B6.129P2(C)-Mecp2^tm1-1Bird^ (Guy et al., 2001) maintained on a C57BL/6J background was obtained from The Jackson Laboratory (Bar Harbor, ME, USA). Mecp2 knockout males (*Mecp2^-/y^*) were generated by crossing hemizygous *Mecp2^+/-^* females with C57BL/6J wild type males. Animals were kept in a temperature- und humidity-controlled 12 h light-dark cycle and had free access to water and standardized pellet food.

### Genotyping

The genotype of newborn mice was determined using PCR on DNA isolated from mice tail biopsies. Mice tails were incubated in 25 mM NaOH/0.2 mM EDTA for 3 h at 65°C. After neutralization with equal volume of 40 mM Tris/HCl pH 5.5, 1 μl was used as template in a subsequent PCR. Primers for genotyping are given in **Table 1**.

**Table 1:** List of primers used in this study for genotyping, cloning and quantitative RT-PCR.

*Genotyping*	*Mecp2^-/y^* WT	*for*	GACCCCTTGGGACTGAAGTT	NM_010788.3
		*rev*	CCACCCTCCAGTTTGGTTTA	
	*Mecp2^-/y^* KO	*for*	CCATGCGATAAGCTTGATGA	
		*rev*	CCACCCTCCAGTTTGGTTTA	

*Cloning*	*Htr5b full length*	*for*	ATGGAAGTTTCTAACCTCTC	NM_010483.3
		*rev*	TTATCTCTGCTTAGTAAAGAG	
	*Htr5b trunc*	*for*	ATGATCGCGATCACCTGGG	
		*rev*	TTATCTCTGCTTAGTAAAGAG	

*RT-qPCR*	*Htr1a*	*for*	AACTATCTCATCGGCTCCTT	NM_008308.4
		*rev*	GATTGCCCAGTACCTGTCTA	
	*Htr1b*	*for*	CTCCATCTCTATTTCGTTGC	NM_010482.1
		*rev*	GTCTTGTTGGGTGTCTGTTT	
	*Htr1d*	*for*	CCATCCATCTTGCTCATTAT	NM_008309.4
		*rev*	CACCTGGTTGAAAAAGAGAG	
	*Htr1f*	*for*	TTTCTACATCCCGCTTGTAT	NM_008310.3
		*rev*	TCGGACAAGGATTTTTCTAA	
	*Htr2a*	*for*	TGTGATGCTTTTAACATTGC	NM_172812.2
		*rev*	CCAACTTACTCCCATGCTAC	
	*Htr2b*	*for*	GAACATCCTTGTGATTCTGG	NM_008311.2
		*rev*	AGGCAGTTGAAAAGAGAACA	
	*Htr2c*	*for*	CTATTTTCAACTGCGTCCAT	NM_008312.4
		*rev*	ATTCACGAACACTTTGCTTT	
	*Htr3a*	*for*	TGGTCCTAGACAGAATAGCC	NM_013561.2
		*rev*	GGTCTTCTCCAAGTCCTGA	
	*Htr4*	*for*	CCTCACAGCAACTTCTCCTT	NM_008313.4
		*rev*	TCCCCTGACTTCCTCAAATA	
	*Htr5a*	*for*	TGCTCTTTGTGTACTGGAAA	NM_008314.2
		*rev*	ACGTATCCCCTTCTGTCTG	
	*Htr5b*	*for*	GAGTCTGAGATGGTGTTCA	NM_010483.3
		*rev*	AATATCCAAGCCACAGGAAT	
	*Htr6*	*for*	CTGAGCATGTTCTTTGTCAC	NM_021358.2
		*rev*	CATGAAGAGGGGATAGATGA	
	*Htr7*	*for*	GTTAGTGTCACGGACCTCAT	NM_008315.2
		*rev*	ATCATTTTGGCCATACATTT	
	*Slc6a4*	*for*	AAGCCAAGCTGATGATGTAA	NM_010484.2
		*rev*	TCCTCACATATCCCAGTCAG	
	*Ddc*	*for*	GCAGTGCCTTTATCTGTCCT	NM_001190448.1
		*rev*	GAATCCTGAGTCCTGGTGAC	
	*Tph2*	*for*	CAGGGTCGAGTACACAGAAG	NM_173391.3
		*rev*	CTTTCAGAAACATGGAGACG	
	*Hprt*	*for*	ATTAGCGATGATGAACCAGG	NM_013556.2
		*rev*	GTCAGCAAAGAACTTATAGCCC	
	*Gapdh*	*for*	CAAGCTCATTTCCTGGTATGAC	NM_008084.1
		*rev*	AGGCCCCTCCTGTTATTATG	


*Accession numbers are given for the murine gene used to generate the primers. If there were multiple transcript variants of a gene, our primers were designed to be specific for the main transcript and did not discriminate between transcript variants.*

### Quantitative RT-PCR

Gene expression was analyzed by quantitative RT-PCR analysis. The total ribonucleic acid (RNA) of homogenized brain tissue (hippocampus and brainstem) was isolated using the Trizol^®^ method according to manufacturer’s instructions (GibcoBRL) and its concentration was determined using the nanodrop ND-1000 spectrophotometer followed by its quality and integrity measurement by electrophoresis on RNA 6000 LabChip^®^ kit (Agilent 2100 Bioanalyzer). The RNA was transcribed into the corresponding deoxyribonucleic acid (cDNA) using the iScript cDNA Synthesis Kit (BioRad). The primer pairs (**Table 1**) were designed by using the Primer3 program^1^.

Gel electrophoresis revealed a single polymerase chain reaction (PCR) product, and the melting curve analysis showed a single peak for all amplification products. The PCR products were sequenced and blasted to confirm the correct identity of each amplicon. Ten-fold serial dilutions generated from cDNA of each sample were used as a reference for the standard curve calculation to determine primer efficiency. Real-time PCR reactions (25 μl) were performed in triplicates containing 1/20 volume of the sample cDNA preparation from 250 ng total RNA, 400 nM of each primers, and 1X iQ-SYBR Green Supermix (BioRad, Laboratories, Ltd). The PCR-reactions were performed as follows: initial denaturation at 98°C for 30 s, 40 cycles of (denaturation 94°C/1 s, annealing 58°C/15 s, extension 72°C/1 s), and a final gradual increase of 0.5°C in temperature from 60°C to 90°C. All real-time quantifications were performed using the iCycler iQ system (BioRad) and were adjusted by using the method according to (Pfaffl, 2001). Hypoxanthine guanine phosphoribosyl transferase (*Hprt*) served as reference gene (“housekeeping gene”) for normalization in the qPCR.

### Cell Culture, Transfection and Plasmids

Murine neuroblastoma cell line N1E-115 was obtained from the American Type Culture collection (ATCC). Cells were grown in Dulbecco’s modified Eagle’s medium (DMEM) containing 10% fetal calf serum (FCS) and 1% penicillin/streptomycin at 37°C under 5% CO_2_.

For transient transfection, cells were seeded in cell culture dishes and transfected with indicated plasmids using Lipofectamine2000 Reagent (Invitrogen) according to the manufacturer’s instruction.

Serotonin receptor 5b (5-ht_5b_) expression constructs were generated from murine cDNA. Brain tissue was explanted and used for total RNA isolation with the OLS RNA kit (OLS, Germany) according to the manufacturer’s instructions. The total RNA was used in one-step RT-PCR (Invitrogen) and resulting PCR fragment was cloned into pTarget expression vector (Promega). Primer sequences are given in **Table 1**. To obtain a C-terminal fluorophore fusion construct of 5-ht_5b_, the receptor and the fluorophores CFP, GFP, YFP or mCherry were amplified individually, and fusion PCR was used to combine the cDNAs. The resulting PCR fragment was cloned into pTarget expression vector (Promega). To test the hypothesis of a truncated 5-ht_5b_ receptor, we amplified the unlabeled and the mCherry fusion construct and cloned the fragments into pTarget. Correct sequences of all constructs were determined by sequencing.

The G_i3α_-CFP plasmid was kindly provided by Dr. Andrew Tinker (University College, London, UK). The G_sα_-GFP plasmid was a kind gift of Dr. Mark Rasenick (U. Illinois College of Medicine, Chicago).

### Counterstaining of Intracellular Compartments

The plasmids to fluorescently label the cell membrane (pYFP-Mem), the endoplasmic reticulum (pYFP-ER), mitochondria (pYFP-mito) and peroxisomes (pEGFP-Pex) were obtained from Clontech. The plasmid to label the endosomes (GFP-Rab5; Addgene plasmid # 31733) was a gift from Richard Pagano (Choudhury et al., 2002). Lysosomes were counterstained with LysoTracker (Thermo Scientific). The golgi apparatus was counterstained using antibodies directed against *cis* golgi marker GM-130 (sc-55591, Santa Cruz) and trans-golgi marker TGN38 (sc-166594, Santa Cruz).

Co-localization was observed using Zeiss LSM 510 Meta system. Quantitative analysis of co-localization was carried out by calculating Pearson’s correlation coefficients using LSM 510 software.

### Immuno-Staining Procedures

#### Preparation of Tissue

To obtain tissue for immuno-fluorescence analysis, wild type and *Mecp2^-/y^* mice (P40) were deeply anesthetized with isoflurane (1-Chloro-2,2,2-trifluoroethyl-difluoro-methylether, Abbott, Germany) until they were unresponsive to pain stimuli. A thoracotomy was performed and animals were transcardially perfused with 50 ml of 0.9% NaCl followed by 200 ml of 4% phosphate-buffered formaldehyde (10 ml/min). The brain was removed and post-fixed for 4 h with the same fixative at 4°C. Whole brains were stored in 1% formaldehyde in PBS at 4°C. Before sectioning, brains were equilibrated in HEPES buffer (7.5 g NaCl, 0.3 g KCl, 0.06 g KH_2_PO_4_, 0.13 g Na_2_HPO_4_, 2 g Glucose, 2.4 ml 10 mM HEPES, 0.1 g MgCl_2_, 0.05 g MgSO_4_, 0.165 g CaCl_2_, pH 7.4) for 48 h, cryoprotected in 15% sucrose in PBS for 24 h followed by equilibration in 30% sucrose in PBS for 24 h at 4°C, and then frozen at -80°C. Series of 30-μm-thick brain sections ranging from cervical spinal cord to midbrain colliculi were cut using a freezing microtome (Frigocut, Reichert-Jung, Germany). Sections were stored in HEPES buffer. All buffers were supplemented with small amount sodium azide.

#### Generation of Anti-5ht_5b_ Antibodies

The polyclonal antibodies against the mouse 5-ht_5b_ receptor were generated by immunizing three New Zealand White rabbits (Charles River) with a 15mer peptide representing the C-terminus of the mouse 5-ht_5b_ receptor amino acid sequence (NP_034613.1; NH2-KNYNNAFKSLFTKQR-COOH). For immunization purposes, peptides were coupled to keyhole limpet hemocyanin (KLH). The rabbits were immunized with 300 μg KLH-coupled peptide in Hunter’s adjuvant (TiterMax Gold, Sigma) five times (28-days-intervall). After estimation of the antibody titer using enzyme-linked immune-sorbent assay (ELISA) with solid phase-coated peptide, antibodies were purified on an antigen-coupled CNBr-activated Sepharose^®^ 4B column. The eluate was dialyzed against two changes of 5 l PBS for 24 h at 4°C, and finally concentrated to at least 1 μg IgG/μl.

#### Immuno-Fluorescence Microscopy

Immuno-fluorescence analysis was started with antigen retrieval using citrate buffer (10 mM citric acid, 0.05% Tween20, pH 6.0) at 80°C for 30 min. Sections were incubated in blocking buffer (PBS, 0.1% Triton-X100, 1% Tryptone/Peptone) for 60 min at RT to permeabilize and block non-specific binding. Primary antibody (rabbit anti-5ht_5b_) was diluted 1:100 in blocking buffer and incubated for 60 min at RT. After washing in washing buffer (PBS, 0.05% Tween20, 0.3% Triton X100), sections were incubated for 1 h at RT in the dark with anti-rabbit atto647-conjugated secondary antibodies (Sigma–Aldrich, Cat. No. 40839) diluted 1:400 in blocking buffer. Sections were counterstained with DAPI during the wash step, then mounted onto microscope-slides and cover-slipped with Mowiol. Immuno-fluorescence was analyzed with a Zeiss fluorescence microscope (Zeiss, Germany). Images were taken at 10x magnification and were imported into ImageJ (Schneider et al., 2012), digitally adjusted if necessary for brightness and contrast, and assembled into plates. All buffers were supplemented with small amount sodium azide.

#### Immunoelectron Microscopy

Immunoelectron microscopy of ultrathin cryosections was performed as described previously (Feldmann et al., 2011). In brief, WT and MeCP2-deficient mice were anesthetized with isoflurane and transcardially perfused with 4% formaldehyde (Serva) in 0.1 M phosphate buffer. Vibratome sections of the brain stem were infiltrated with 2.3 M sucrose in 0.1 M phosphate buffer overnight. Small blocks from the region of the brainstem were mounted onto aluminum pins for ultramicrotomy and frozen in liquid nitrogen. Ultrathin cryosections were immune-labeled with rabbit antibodies specific for 5-ht_5b_ receptor and protein A-gold (10 nm) obtained from the Cell Microscopy Center, Department of Cell Biology, University Medical Center Utrecht, The Netherlands. Sections were analyzed with a LEO EM912AB (Zeiss, Oberkochen) and digital micrographs were obtained with an on-axis 2,048 × 2,048-CCD camera (TRS, Moorenweis).

#### Western Blotting of the 5-ht_5b_ Receptor

Brain tissue from *Mecp2^-/y^* mice or N1E-115 cells transfected with expression plasmids encoding the full-length murine 5-ht_5b_ receptor were resuspended in 300 μl Laemmli buffer [20 mM Tris/HCl, pH 6.8, 2 mM EDTA, 2% (w/v) SDS, 10% (v/v) 2-mercaptoethanol, 10% (v/v) glycerol and 0.3% (w/v) bromophenol blue] supplemented with a protease inhibitor cocktail (Sigma) and boiled for 5 min at 95°C. Proteins (50 μg of each sample) were separated using 10% SDS–PAGE and transferred onto a nitrocellulose membrane. The membrane was blocked with 2% w/v BSA/TBS (pH 7.4) for 30 min at RT and antigen was detected using a primary polyclonal antibody (1:1,000 dilution) for 120 min at RT. Secondary antibodies (IRDye 800CW-conjugated anti-rabbit, LI-COR, Lincoln, NE, USA) were used at a dilution of 1:10,000 for 2 h at RT. The visualization of the antigen–antibody reaction was performed using the Odyssey detection system (LI-COR). To test specificity, the anti-5-ht_5b_ receptor antibody was pre-incubated with a 50-fold molar excess of the immunizing peptide, which led to the vanishing of the specific 22.5 kDa band.

### Assay for [35S]GTPγS Binding and Immune-Precipitation of G Protein α Subunits

Membrane preparations of transiently transfected mouse N1E-115 neuroblastoma cells expressing the full-length or truncated 5-ht_5b_ receptor linked to cherry fluorophore and G-protein α subunits (G_αi3_ and G_αs_ fused to variants of green fluorescent protein) were performed according to the protocol described by Kvachnina et al. (2009). Membrane preparations were diluted in reaction buffer (50 mM Tris/HCl pH 7.4, 2 mM EDTA, 100 mM NaCl, 3 mM MgCl_2_, and 1 μM GDP) to a concentration of 1 μg/μl. Each reaction contained 50 μg membranes in a total volume of 100 μl. Reactions were performed in triplicate. After adding [35S]GTPγS (Hartmann Analytic) to a final concentration of 3 nM, samples were incubated for 5 min at 30°C in the presence or absence of 1 μM 5-HT. The reaction was terminated by adding 900 μl of RIPA-buffer (20 mM Tris/HCl pH 7.4, 0.15 M NaCl, 10 mM EDTA, 10 mM Iodacetamide, 1% Triton X-100, 1% sodium deoxycholate and 0.1% SDS) for 30 min on ice. Samples were centrifuged at 13,000 rpm for 10 min. The supernatants were transferred into a new tube and samples were incubated for 2 h after addition of 100 μl protein A-Sepharose (Sigma, 20 mg/ml RIPA) and 0.5 μl of goat anti-GFP antibodies (University of Alberta, TEC Edmonton). Immuno-precipitates were washed three times, boiled in 0.5 ml of 0.5% SDS, and radioactivity was measured by scintillation counting.

### Statistical Analysis

Statistical significance of the data was tested by non-parametric Mann–Whiney test using GraphPad Prism 5 (GraphPad Software Inc., La Jolla, CA, USA) on a Microsoft Windows PC. *P*-value less than 0.05 is considered significant. Data are presented as mean ± standard error of the mean (SEM).

## Results

### The Serotonergic System

We used quantitative RT-PCR to analyze the complete serotonergic system in hippocampus and brainstem of WT and *Mecp2^-/y^* mice on the transcriptional level. We analyzed a total of 16 genes that included the serotonin receptors *Htr1a*, *Htr1b*, *Htr1d*, *Htr1f*, *Htr2a, Htr2b, Htr2c*, *Htr3a*, *Htr4*, *Htr5a*, *Htr5b*, *Htr6*, *Htr7*, the serotonin transporter (*Slc6a4*), and the synthesizing enzymes dopamine decarboxylase (*Ddc*) and tryptophan hydroxylase 2 (*Tph2*). The serotonin receptor *Htr1e* was not analyzed as it is absent in mice. Also note that *Htr1c* is not listed above. The gene was, after its initial discovery and characterization, renamed to *Htr2c* (OMIM 312861). **Table 1** lists the accession numbers of the murine genes of the serotonergic system and the sequences of the primers used for RT-qPCR.

Analyses were performed at postnatal day 40 (P40), a stage where *Mecp2^-/y^* mice have already developed a RTT phenotype with hind limb clasping, low body weight and several other of the mentioned symptoms (Stettner et al., 2008). All differentially expressed genes are summarized in **Table 2**.

**Table 2:** Dysregulated genes of the serotonergic system in brainstem and hippocampus at P40.

	Hippocampus	Brainstem
*Htr1d*	–	↑ (^∗∗^)
*Htr2c*	–	↓ (^∗∗^)
*Htr5b*	–	↑ (^∗∗^)
*Ddc*	↑ (^∗∗^)	↑ (^∗∗∗^)
*Slc6a4*	↓ (^∗∗∗^)	–
*Tph2*	–	↓ (^∗∗^)


*Asterisks indicate significance (^∗∗^*p* ≤ 0.01; ^∗∗∗^*p* ≤ 0.001).*

### Regulation of Components of the Serotonergic System in the Brainstem

**FIGURE 1:**
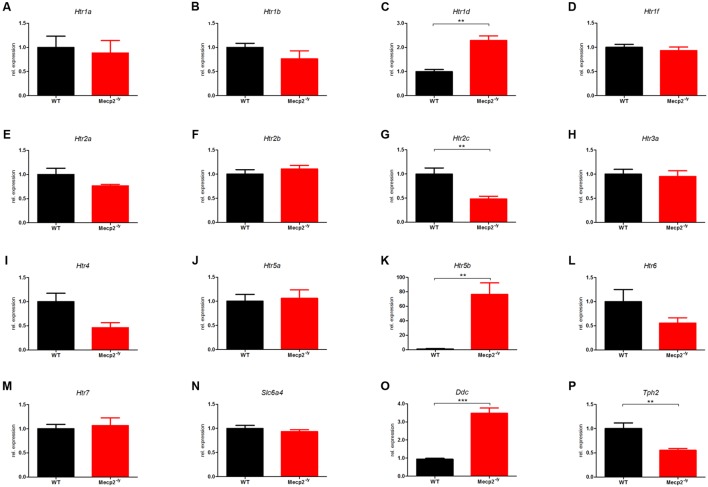
**Comparison of mRNA expression of the serotonergic system in WT vs. *Mecp2^-/y^* mice in the brainstem at P40.** Messenger RNA level in the brainstem was measured by qPCR for WT (black) and Mecp2^-/*y*^ (red) mice at postnatal day 40. **(A)** *Htr1a*, **(B)** *Htr1b*, **(C)** *Htr1d*, **(D)** *Htr1f*, **(E)** *Htr2a*, **(F)** *Htr2b*, **(G)** *Htr2c*, **(H)** *Htr3a*, **(I)** *Htr4*, **(J)** *Htr5a*, **(K)** *Htr5b*, **(L)** *Htr6*, **(M)** *Htr7*, **(N)** *Slc6a4*, **(O)** *Ddc*, and **(P)** *Tph2*. The bar diagrams represent the results of separate qPCR analyses with mean values and standard error of the mean from *n* = 5 biological replicates of genes for the serotonin receptors *Htr1a*, *Htr1b*, *Htr1d*, *Htr1f*, *Htr2a, Htr2b, Htr2c*, *Htr3a*, *Htr4*, *Htr5a*, *Htr5b*, *Htr6*, *Htr7*, the serotonin transporter (*Slc6a4*), and the enzymes dopamine decarboxylase (*Ddc*) and tryptophan hydroxylase 2 (*Tph2*). The *y*-axis shows relative expression. Asterisks indicate significance (^∗∗^*p* ≤ 0.01; ^∗∗∗^*p* ≤ 0.001).

The differential expression levels for all 16 components of the murine serotonergic system in the brainstem are given in **Figure 1**, where the WT was set to 1. In the brainstem, 5 of the 16 genes of the murine serotonergic system were dysregulated between WT and *Mecp2^-/y^* mice at P40. These were the three serotonin receptor genes *Htr1d*, *Htr2c* and *Htr5b*, as well as the non-receptor genes *Ddc* and *Tph2*. Also, *Htr4* and *Htr6* showed strong, although not yet significant dysregulation.

The mRNA level for *Htr1d* in *Mecp2^-/y^* mice showed a 2-fold up-regulation (**Figure 1C**; 2.29 ± 0.184, *p* = 0.0013). *Htr1d* affects locomotion and anxiety and also induces vascular vasoconstriction in the brain. In addition, 5-HT_1D_R has been implicated in controlling activity of raphé neurons and therefore serotonin release. An up-regulation of 5-HT_1D_R in the brainstem could therefore inhibit the serotonin release in the raphé nuclei and thus explain the low brain serotonin levels reported previously (Mokler et al., 1998).

The mRNA level of *Htr2c* was reduced to roughly half compared with WT (**Figure 1G**; 0.482 ± 0.057; *p* = 0.005). *Htr2c* activation inhibits dopamine and norepinephrine release in the striatum, prefrontal cortex, nucleus accumbens, hippocampus, hypothalamus, and amygdala among other areas (Alex et al., 2005). It also regulates mood, anxiety, feeding, and reproductive behavior (Heisler et al., 2007). In the brainstem, *Htr2c* is associated with respiratory dysfunction (Tecott et al., 1995; Hodges et al., 2009).

The most striking difference was observed for *Htr5b* with an up-regulation in *Mecp2^-/y^* mice by a factor of 75 at P40 compared to age-matched WT mice (**Figure 1K**; 76.43 ± 15.86; *p* = 0.0089). *Htr5b* showed a tremendous dysregulation with nearly 75-fold upregulation in *Mecp2^-/y^* mice. Unfortunately, little is known about this specific serotonin receptor. Based on molecular studies, *Htr5b* is grouped with *Htr5a* forming the fifth class of serotonin receptors. The two 5-ht_5_ receptors are the only serotonin receptors that are transcribed from two exons (Matthes et al., 1993). *Htr5b* is believed to be non-functional in humans because its coding sequence is interrupted by several stop codons and repeated insertions (Grailhe et al., 2001), while rodents carry a fully intact *Htr5b* gene. Therefore, we performed some basic tests to determine the relevance of *Htr5b* dysregulation in RTT (see below).

Tryptophan hydroxylase 2 (*Tph2*) was reduced 2-fold in *Mecp2^-/y^* mice compared to WT (**Figure 1P**; 0.553 ± 0.379, *p* = 0.0067). *Tph2* is the main synthesizing enzyme for serotonin, hydroxylating the rate-limiting step from L-tryptophan to 5-hydroxy-L-tryptophan (Kuhar et al., 1999). *Tph2* is mainly expressed in neurons of the raphé nuclei, which contain the majority of serotonin-containing neurons (Frazer and Hensler, 1999). A defect of *Tph2* could explain reduced levels of serotonin in the brain and in the cerebrospinal fluid (Jellinger, 2003; Ramaekers et al., 2003). The dopamine decarboxylase (*Ddc*) level was increased 3-fold in *Mecp2^-/y^* mice (**Figure 1O**; 3.48 ± 0.299, *p* < 0.001).

### Regulation of Components of the Serotonergic System in Hippocampus

**FIGURE 2:**
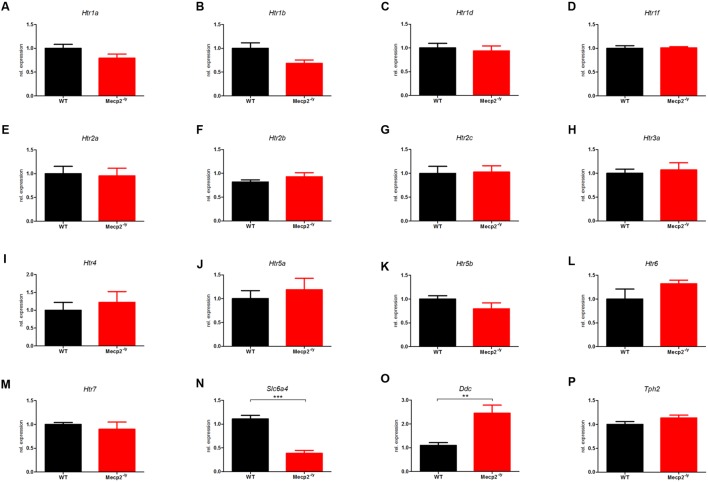
**Comparison of mRNA expression of the serotonergic system in WT vs. *Mecp2^-/y^* mice in the hippocampus at P40.** Messenger RNA level in the hippocampus was measured by qPCR for WT (black) and *Mecp2^-/y^* (red) mice at postnatal day 40. **(A)** *Htr1a*, **(B)** *Htr1b*, **(C)** *Htr1d*, **(D)** *Htr1f*, **(E)** *Htr2a*, **(F)** *Htr2b*, **(G)** *Htr2c*, **(H)** *Htr3a*, **(I)** *Htr4*, **(J)** *Htr5a*, **(K)** *Htr5b*, **(L)** *Htr6*, **(M)** *Htr7*, **(N)** *Slc6a4*, **(O)** *Ddc*, and **(P)** *Tph2*. The bar diagrams represent the results of separate qPCR analyses with mean values and standard error of the mean from *n* = 5 biological replicates of genes for the serotonin receptors *Htr1a*, *Htr1b*, *Htr1d*, *Htr1f*, *Htr2a, Htr2b, Htr2c*, *Htr3a*, *Htr4*, *Htr5a*, *Htr5b*, *Htr6*, *Htr7*, the serotonin transporter (*Slc6a4*), and the enzymes dopamine decarboxylase (*Ddc*) and tryptophan hydroxylase 2 (*Tph2*). The *y*-axis shows relative expression. Asterisks indicate significance (^∗∗^*p* ≤ 0.01; ^∗∗∗^*p* ≤ 0.001).

The differential expression levels for all 16 components of the murine serotonergic system in the hippocampus are given in **Figure 2**, where the WT was set to 1. In hippocampus, of the 16 genes of the serotonergic system, only two showed differential expression between WT and Mecp2^-/*y*^ mice.

*Ddc*, which was upregulated threefold in the brainstem, was also up-regulated twofold in hippocampus (**Figure 2O**; 2.459 ± 0.336, *p* = 0.0015). *Ddc* catalyzes several different decarboxylation reactions (Siegel et al., 1999), thus mutations in *Ddc* lead to reduced levels of neurotransmitters dopamine and serotonin (Ramaekers et al., 2003). However, neither for dopamine nor for serotonin synthesis, *Ddc* is the rate-limiting enzyme (Kuhar et al., 1999), whereas in humans *Ddc* is the rate-limiting enzyme for the synthesis of trace amine transmitter (Berry, 2004). For the serotonergic system, *Ddc* catalyzes the decarboxylation of 5-Hydroxy-L-Tryptophan to serotonin, while *Ddc* also catalyzes the conversion of 3,4-dihydroxyphenylalanine (DOPA) and Levodopa to dopamine. Dopamine in the basal ganglia is important for coordination and smooth movement, and *Mecp2^-/y^* mice show disturbed motor function like hind limb clasping.

The second dysregulated gene in hippocampus was *Slc6a4*) which was down-regulated in *Mecp2^-/y^* mice (**Figure 2N**; 0.387 ± 0.611, *p* < 0.001). *Slc6a4* (solute carrier family 6 member 4) encodes the serotonin transporter SERT (Blakely et al., 1991; Hoffman et al., 1991) and removes serotonin from the synaptic cleft, thus terminating its action by transporting 5-HT into the presynaptic neuron in a sodium-dependent manner (Horschia, 2001). Mutations in the *Slc6a4* promoter affect the rate of serotonin uptake (Caspi et al., 2003) and are therefore associated with numerous diseases like sudden infant death syndrome (Weese-Mayer et al., 2003), and propensity to post-traumatic stress disorder (Liu et al., 2015) or susceptibility for depression (Lin et al., 2015).

An up-regulation of *Ddc* in RTT would have little effect on the availability and therefore on the strength of synaptic transmission. However, a down-regulation of *Slc6a4* in RTT would result in increased serotonin-mediated synaptic transmission due to longer dwell time of 5-HT in the synaptic cleft. Therefore, and because no other genes were found dysregulated in hippocampus, we believe that upregulation of *Ddc* and down-regulation of *Slc6a4* could be an adaption to low serotonin levels reported previously (Ramaekers et al., 2003).

### Differences between Brainstem and Hippocampus

In our mouse model of RTT syndrome, 6 out of 16 genes constituting the serotonergic system in mouse were differentially regulated. Of these six genes, only two genes were dysregulated in hippocampus and only dopamine decarboxylase (*Ddc*) was dysregulated in both brain regions. *Ddc* was up-regulated in *Mecp2^-/y^* mice in hippocampus and brainstem by approximate the same factor, which might suggests a genetic reason for the up-regulation, e.g., the missing of transcriptional inhibitor *Mecp2*. Nevertheless, in hippocampus, up-regulation of *Ddc* might compensate for the down-regulation of *Slc6a4* or vice versa. There have been reports of reduced serotonin (Jellinger, 2003; Ramaekers et al., 2003) and dopamine (Riederer et al., 1986; Lekman et al., 1989; Wenk et al., 1991) levels in the brain and cerebrospinal fluid of RTT patients which are compensated by increased expression of dopamine receptor (Chiron et al., 1993). The decrease of both neurotransmitters make it like that the dysregulation of both *Ddc* and *Slc6a4* in hippocampus is compensatory.

### Profiling Developmental Changes of *Htr5b*

**FIGURE 3:**
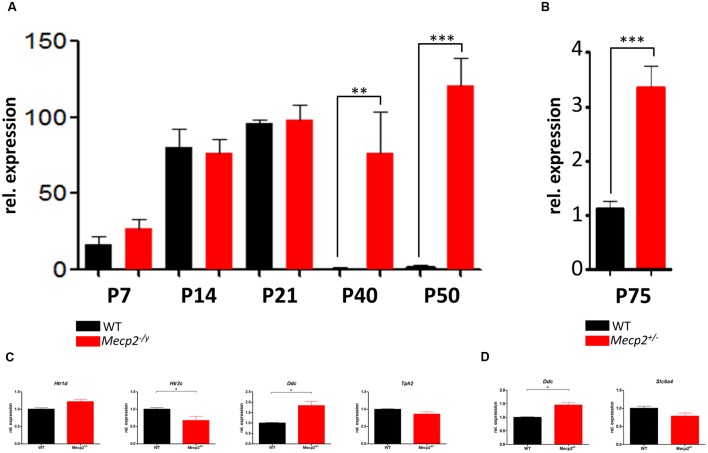
**Developmental profile of *Htr5b* in brainstem of WT and *Mecp2-deficient* mice.** **(A)** Shown are the messenger RNA levels of *Htr5b* at post-natal days P7, P14, P21, P40, and P50 for male WT (black) and *Mecp2^-/y^* (red) mice. *Htr5b* expression increases in parallel in WT and *Mecp2^-/y^* until P21, after which it is downregulated completely in WT, but remains at the previously seen level in *Mecp2^-/y^* mice until P50, indicating a failure of down-regulation. **(B)** Messenger RNA levels of *Htr5b* at post-natal day P75 in female WT and *Mecp2^+/-^* mice. *Htr5b* also displays a significant dysregulation at P75 in female mice, although the dysregulation is by far not as pronounced as in male mice. Analyses were performed in triplicates with at least 3 different animals. **(C)** Messenger RNA levels of *Htr1d, Htr2c, Ddc* and *Tph2* in brainstem at post-natal day P75 in female WT and *Mecp2^+/-^* mice. *Htr1d, Htr2c, and Ddc* in brainstem show a significant dysregulation. **(D)** Messenger RNA levels of *Ddc* and *Slc6a4* in hippocampus at post-natal day P75 in female WT and *Mecp2^+/-^* mice. Analyses were performed in triplicates with at least 3 different animals. The *y*-axis shows relative expression. Asterisks indicate significance (^∗^*p* ≤ 0.05; ^∗∗^*p* ≤ 0.01; ^∗∗∗^*p* ≤ 0.001).

After identifying *Htr5b* to be profoundly dysregulated in the brainstem at P40, we profiled the expression of this gene during the development at P7, P14, P21, P40, and P50 (**Figure 3A**). Up until P21, *Htr5b* expression increases uniformly in WT and *Mecp2^-/y^* mice. After reaching peak expression around P21, *Htr5b* expression in WT mice is essentially turned off after P21. However, in *Mecp2^-/y^* mice it remains at the same elevated levels. Thus, the observed dysregulation is not due to an upregulation of *Htr5b* in *Mecp2^-/y^* mice, but due to a failure to down-regulate *Htr5b* expression. In hemizygous female mice at P75, we also found a significant dysregulation, albeit not as strong as in male knockout mice (**Figure 3B**). However, female mice at this age do not display the same advanced disease progression as male mice and they are mosaics, displaying varying gene expression patterns. Considering the identical profiles in WT and *Mecp2^-/y^* mice up to P21, one can speculate that *Htr5b* expression may serve a physiological purpose in mice. The intracellular localization of 5-ht_5b_ and the nearly complete down-regulation after P21 indicates that 5-ht_5b_ might act more like a regulator than a receptor. In addition to *Htr5b*, we also assayed the other genes found dysregulated in P40 male mice again in P75 female mice (**Figures 3C,D**). All genes showed a comparable trend in the female mice as found in the male mice, although the differences between WT and *Mecp2^+/-^* animals were much smaller. In brainstem (**Figure 3C**), *Htr2c* (0.672 ± 0.124, *p* = 0.012) and *Ddc* (1.841 ± 0.198, p = 0.026) were found to be significantly dysregulated. In hippocampus (**Figure 3D**), only *Ddc* showed a significant dysregulation (1.452 ± 0.1, *p* = 0.0286). The lower number of significantly dysregulated genes as well as the smaller differences between WT and *Mecp2^+/-^* females could be explained in two ways. Hemizygous mice are a mosaic regarding *Mecp2* activity, thus generating greater variability of gene regulation. Also, females develop symptoms much later, so possible differences may not be detectable at P75.

### Molecular Analysis of Serotonin Receptor 5b (5-ht_5b_)

The tremendous dysregulation of *Htr5b* and lack of previous research prompted us to analyze this receptor in more detail at the protein level.

**FIGURE 4:**
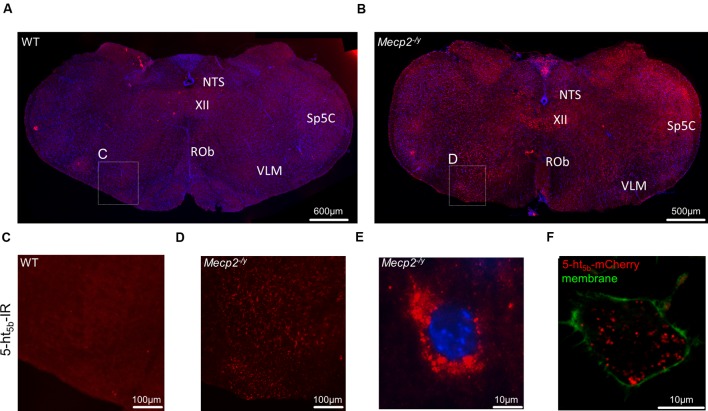
**Distribution and upregulation of 5-ht_5b_ in brainstem of *Mecp2^-/y^* mice.** Representative immunohistological stainings of one out of three animals analyzed each. **(A)** In WT mice, 5-ht_5b_-receptor-positive cells (red) were virtually undetectable at postnatal day 40 (P40). **(B)** In contrast, mice with MeCP2 deficiency (*Mecp2^-/y^*) displayed a marked increase in the number of 5-ht_5b_-receptor immuno-reactive cells. **(C,D)** Higher magnification images of areas indicated in **(A,B)**. **(E)** High magnification images of 5-ht_5b_-receptor-positive cells (red) revealed a clustered intracellular distribution indicating organelle localization of receptors. **(F)** The same clustered intracellular distribution was found when full length *Htr5b* fused to mCherry was expressed in murine neuroblastoma cell line N1E-115. Nuclei are counterstained with DAPI (blue). NTS, nucleus of the solitary tract; Rob, raphe obscurus nucleus; Sp5C, spinal trigeminal nucleus, caudal part; VLM, ventrolateral medulla; XII, hypoglossal nucleus. Scale bars as indicated.

If any, only weak 5-ht_5b_ receptor expression was found in the hippocampus of both WT and *Mecp2^-/y^* mice at P40 (data not shown). The same was true for the brainstem of WT mice (**Figures 4A,C**), while in MeCP2-deficient mice, 5-ht_5b_ receptors were found in abundance (**Figures 4B,D**). Thus, receptor staining was in agreement with our qPCR expression data. Closer inspection of the immune-histochemical data revealed an unusual staining pattern for 5-ht_5b_ with punctate intracellular pattern instead of uniform labeling of the cytoplasm (**Figure 4E**). Interestingly, transfection of a 5ht_5b_-fluorescent protein fusion construct in neuroblastoma cells produced the same intracellular expression pattern (**Figure 4F**).

**FIGURE 5:**
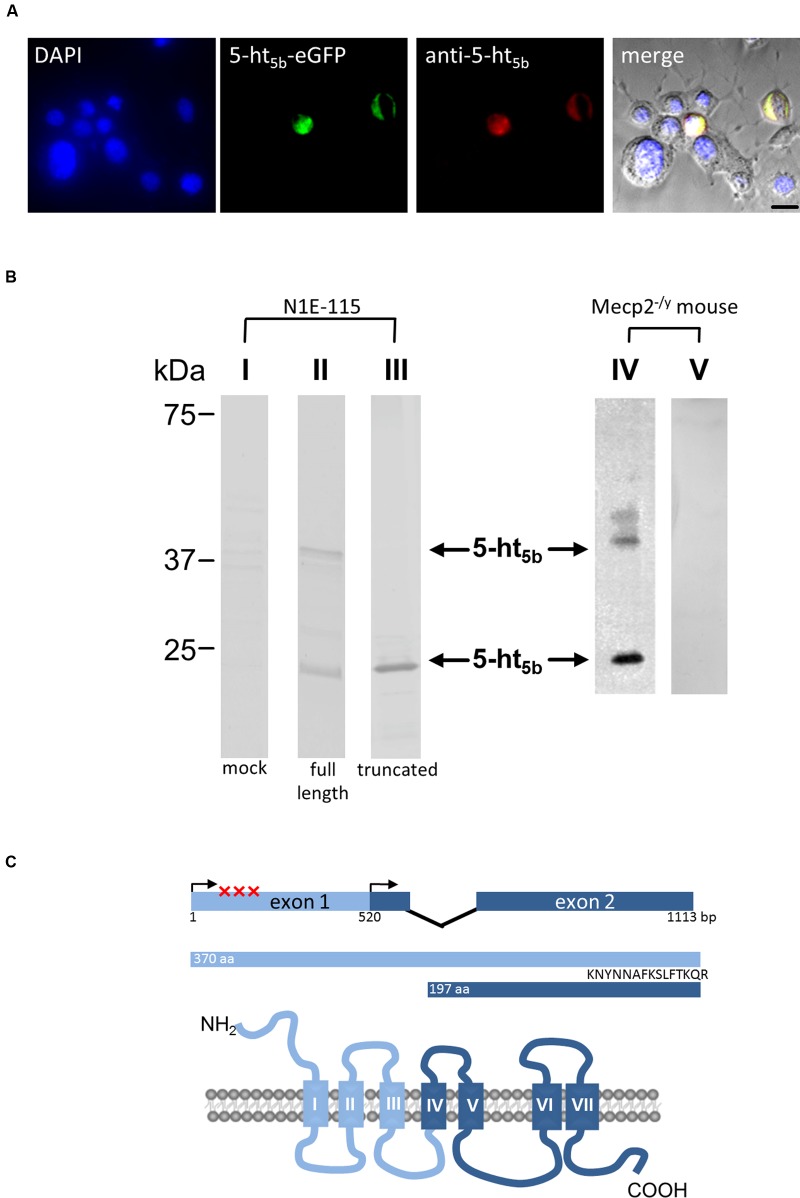
**Molecular analysis of 5-ht_5b_.** **(A)** Specificity control of the antibody used in **(B)** and **Figure 6**. N1E-115 neuroblastoma cells were transfected with an plasmid coding for 5-ht_5b_-eGFP and were counterstained with DAPI (blue) and with the antibody generated against the C-terminus of 5-ht_5b_. The antibody was visualized with an atto647-coupled secondary antibody (red, Sigma–Aldrich, Cat. No. 40839**).** Only cells transfected with 5-ht_5b_-eGFP show reactivity with the antibody. **(B)** Immuno-blots of N1E-115 neuroblastoma cells transfected with expression constructs for unlabeled full-length or truncated 5-ht_5b_ (*Htr5b*-tr) receptor (I – III) and of *Mecp2^-/y^* mouse brainstem lysates (IV, V). While no signal is detectable in mock-transfected cells (I), a signal at about 40 kDa corresponding to the full length protein is detected in transfected cells (II) and murine tissue (IV). Both in cells (II) and murine tissue (IV), an additional signal is visible at about 22.5 kDa. Such a signal corresponds to a theoretical truncated 5-ht_5b_ receptor (see **B**) and can be reproduced in cells transfected with a truncated expression construct (III). Full length and truncated signal disappear in murine tissue if the antibody is pre-incubated with the antigen used for immunization (V). **(C)** Schematic representation of the *Htr5b* gene with exon-intron structure and the 5-ht_5b_ protein with transmembrane domains. In human, the coding sequence is disrupted by insertion and nonsense mutations (marked by X). However, translation from the second ATG might produce a truncated protein of 197 amino acids with a predicted relative molecular mass of 22.5 kDa, corresponding with the smaller signal in Western blot analyses. The peptide used for antibody generation is indicated.

Western blot analyses of both brainstem tissue (**Figure 5B**, IV) and neuroblastoma cells transfected with *Htr5b* (**Figure 5B**, II) verified the presence of a protein of approximately 40 kDa, as expected from the genetic sequence. In addition, western blots of transfected cells and murine tissue revealed a signal at 22.5 kDa (**Figure 5B**, II, IV). Such a signal is also obtained when a truncated 5-ht_5b_ is transfected (**Figure 5**, III). The truncated receptor of 22.5 kDa might be important for *Htr5b* expression in humans. The human *Htr5b* gene is considered to be a pseudo-gene, as its first exon is disrupted by several mutations (Grailhe et al., 2001). However, sequence comparison between different species indicated the presence of a highly conserved second start codon surrounded by a classic Kozak sequence (Grailhe et al., 2001) in *Htr5b*. Transcription starting from this second exon would result in a shortened protein with a predicted molecular mass of 22.5 kDa. An artificially truncated murine 5-ht_5b_ receptor behaved nearly identical to the full length version (data not shown), leaving the possibility that a truncated 5-ht_5b_ might also be present in humans and contributes to RTT. Incidentally, two immune-reactive bands were also reported for 5-ht_5a_ (Dutton et al., 2008). As both class 5 serotonin receptors are transcribed from two exons (Matthes et al., 1993), the presence of two protein variants might be a class-defining feature.

**FIGURE 6:**
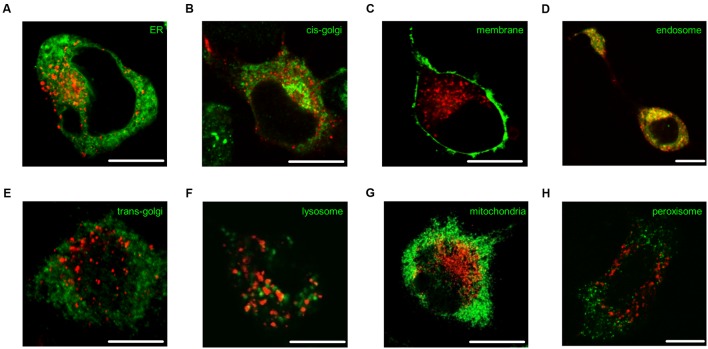
**Subcellular localization of 5-ht_5b_.** Intracellular localization of 5-ht_5b_ receptor (red) counterstained against various compartment markers (green) in N1E neuroblastoma cells: **(A)** endoplasmic reticulum (ER recognition sequence of calreticulin), **(B)** *cis*-golgi network (GM130), **(C)** plasma membrane (GAP-43), **(D)** endosomes (rab5), **(E)** *trans*-golgi network (TGN38), **(F)** lysosomes (LysoTracker) **(G)** mitochondria (mitochondria recognition sequence of cytochrome C-oxidase), and **(H)** peroxisomes (Pex16). Scale bars as indicated.

N1E-115 neuroblastoma cells expressing fluorescent 5-ht_5b_ were either co-transfected with or counter-stained against specific organelle markers to determine 5-ht_5b_’s intracellular compartment. As expected, some co-localization was seen with the ER (**Figure 6A**, correlation coefficient 0.539 ± 0.08) and cis-golgi marker GM130 (**Figure 6B**, 0.584 ± 0.05) as proteins are produced here and are sorted for trafficking to the membrane. However, no receptor reached the plasma membrane (**Figure 6C**, 0.086 ± 0.01). Strong co-localization of 5-ht_5b_ receptors was seen with endosomal compartments, shown exemplarily for early endosome marker rab5 (**Figure 6D**, 0.873 ± 0.02). No co-localization of 5-ht_5b_ receptors was detected with markers for *trans*-golgi marker TGN38 (**Figure 6E**, 0.094 ± 0.03) or with lysosomes (**Figure 6F** 0.359 ± 0.04), meaning that the receptor is neither trafficked to the membrane nor sent immediately for degradation. For completeness, we also checked mitochondria (**Figure 6G**, 0.359 ± 0.04) and peroxisomes (**Figure 6H**, 0.343 ± 0.06), but found no co-localization with 5-ht_5b_.

**FIGURE 7:**
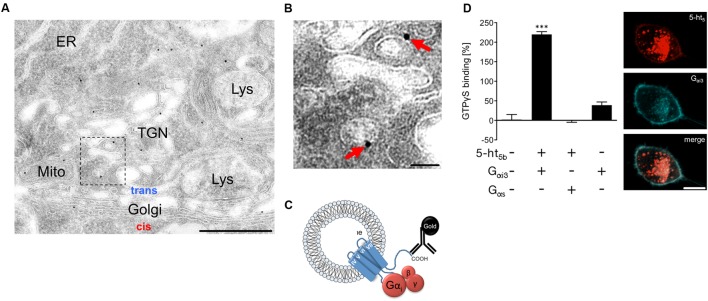
**Signaling mechanisms of intracellular 5-ht_5b_.** **(A,B)** Representative electron microscopic micrograph of a series of tissue sections of the medullary brainstem (*n* = 3 biological replicates) revealed the incorporation of the receptor into organelle membranes. The antibody used was directed against the C terminus, which faces the cytoplasmic site. Scale bars, 500 nm in **(A)** and 50 nm in **(B)**. ER, endoplasmic reticulum; Lys, lysosome; Mito, mitochondrion; TGN, *trans*-Golgi network. **(C)** Suggested organization of the truncated receptor in the membrane of endosomes. The G-protein binding site is accessible from the cytoplasm and the truncation causes a constitutive activity. **(D)** In co-transfected N1E cells, a GTPγS-assay shows binding of GTPγS to 5-ht_5b_ in absence or in presence of G_αi3_ or G_αs_, respectively (*n* = 3; Asterisks indicate significance, ^∗∗∗^*p* < 0.001; one-way ANOVA). Co-expression of 5-ht_5b_ receptor (red) together with inhibitory G_αi3_-protein (blue), produces a faint but visible membrane localization of 5-ht_5b_, which is absent when 5-ht_5b_ is expressed alone (scale bar = 10 μm).

An immune-gold electron microscopic analysis from mouse brainstem revealed that 5-ht_5b_ receptors seemed integrated in vesicular organelles (**Figures 7A,B**), consistent with the light microscopic images (**Figures 6A–H**). The G-protein binding domain of a receptor integrated into organelles would be accessible from the cytoplasm (**Figure 7C**). To determine whether 5-ht_5b_, which has lost its capability to be expressed at the plasma membrane, can still serve its function as a receptor and interact with G-proteins, we studied the signaling pathway of the full-length 5-ht_5b_ receptor in neuroblastoma cells by co-expressing G_αi_ or G_αs_ protein subunits. A GTPγS-assay revealed that cells co-expressing 5-ht_5b_ receptors and G_αi_ proteins exhibited a 220% increase of the baseline signal. Cells co-expressing 5-ht_5b_ receptor and G_αs_ as well as the mock-transfected control did not show any significant changes. The expression of G_αi_ alone resulted in a minor increase of 39% (**Figure 7D**). Overexpression of fluorescently labeled G_αi_ protein led to a faint membrane translocation of otherwise strictly intracellular 5-ht_5b_, indicating protein–protein interaction (**Figure 7D**). Although we found no cell surface expression using recombinant fusion constructs (**Figures 6A–H**), we need to note that early investigations found a reaction of 5-ht_5b_ to stimulation by serotonin in rodents (Matthes et al., 1993), thus a small amount of receptor may reach the cell surface.

## Discussion

The RTT is characterized by cognitive impairment, anxiety, oropharyngeal dysfunctions, cardiac abnormalities, osteopenia, impaired locomotor skills, propensity for seizure and disturbed breathing (Lugaresi et al., 1985; Gillberg, 1986; Glaze et al., 1987; Elian and Rudolf, 1991; Zappella, 1997; Mount et al., 2001; Steffenburg et al., 2001). Most of these symptoms are also present in the *Mecp2* knock-out (*Mecp2^-/y^*) mouse model used here (Guy et al., 2001).

In any animal model of RTT, the choice between heterozygous males or hemizygous females has advantages and disadvantages. Heterozygous females are favored by many, as they might more closely resemble the human situation and therefore better serve translational approaches. However, in these animals the symptoms develop only after months with much broader and milder phenotypes. For gene effect studies like ours, the situation is further complicated by the mosaic-like inactivation of *Mecp2* on the unaffected chromosome. With an early onset of symptoms and lethality starting at P45–50, the MeCP2-deficient male mice might compare better to the early development of symptoms in humans. Most importantly and from an experimental point of view, the complete lack of MeCP2 in this genotype is better suited for the analysis of regulatory effects on the serotonergic system.

We used RT-qPCR to determine the mRNA levels of all 16 components of the murine serotonergic system. In the brainstem, five genes were differentially regulated between WT and *Mecp2^-/y^* mice. The mRNA level of *Htr2c* and *Tph2* were reduced, whereas the mRNA levels for *Htr1d*, *Htr5b* and *Ddc* were increased. In hippocampus, only two genes showed differential expression, with *Slc6a4* being down- and *Ddc* being up-regulated in *Mecp2^-/y^* mice. The down-regulation of two genes seems strange as MeCP2 mainly acts as a transcriptional suppressor (Nan et al., 1997), which absence should lead to an increase of target gene expression. However, there have been reports of MeCP2 also acting as a transcriptional activator (Chahrour et al., 2008). The observed differential expression indicates that the *Mecp2*-dependent (de-) regulation of genes of the serotonergic system is region-specific and suggests different methylation patterns of these genes, since MeCP2 controls gene activity in response to DNA methylation (Nan et al., 1997; Chahrour et al., 2008) and different DNA methylation patterns were reported for other MeCP2-controlled genes (Urdinguio et al., 2008).

The most striking finding was the extent of *Htr5b* dysregulation in the brainstem of *Mecp2^-/y^* mice, which reached up to 75-fold excess at P40 and P50. Interestingly, *Htr5b* dysregulation in symptomatic *Mecp2^-/y^* mice is not caused by early developmental up-regulation, but rather by a failure to down-regulate gene expression at later developmental stages. Moreover, we found that 5-ht_5b_ localizes to intracellular organelles and interacts with G_αi_ proteins. The strongest co-localization of 5-ht_5b_ was with markers for the endosome. This distribution suggests that the receptor does not reach the cell membrane, but does get trapped in intermediate compartments. The abundance and location of 5-ht_5b_ in the brainstem might have potential effects on the physiology of *Mecp2^-/y^* mice. (i) Being still able to interact with G_i_ proteins, the mass of 5-ht_5b_ could scavenge G-proteins, thus blocking G_i_ signaling for other GPCRs. (ii) If the internalized 5-ht_5b_ receptors are constitutively active, changes of the intracellular cyclic adenosine monophosphate (cAMP) concentration would lead to imbalance of the second messenger cascades of many GPCRs. Indeed, such a role of internalized receptors is not uncommon (Irannejad et al., 2013), and constitutively active GPCRs are known for a long time (Tao, 2008). (iii) Moreover, one has to consider the negative effect of 5-ht_5b_ on 5-HT_1A_, which whom it specifically interacts, resulting in reduction of 5-HT_1A_ surface expression (preliminary data; not shown).

The serotonergic system, especially in the brainstem, has been shown to be important for the modulation of breathing (Schwarzacher et al., 2002; Manzke et al., 2010; Niebert et al., 2011). The observed down-regulation of *Htr2c* might, therefore, contribute to the breathing phenotype seen in *Mecp2^-/y^* mice. Interestingly, *Htr2c* knock-out mice have also respiratory arrests with subsequent death following seizures (Tecott et al., 1995). Moreover, the Lmx1bf/f/p mouse strain, which is lacking nearly all serotonergic neurons displayed severe apnea, hypoventilation and reduced hypercapnic response (Hodges et al., 2009).

Although the ultimate cause of the low serotonin levels in RTT patients remains unknown (Mokler et al., 1998), the three genes *Slc6a4*, *Ddc* and *Tph2* could be responsible for this alteration. The tryptophan hydroxylase 2 (*Tph2)* is the rate-limiting enzyme in the synthesis of serotonin (Kuhar et al., 1999) and a downregulation of *Tph2* is in accordance with low brain serotonin levels. Indeed, a reduced *Tph2* expression has been found in MecP2-deficent mice before (Samaco et al., 2009). These results are certainly in accordance with low brain serotonin levels. Nevertheless, the question remains whether reduced *Tph2* expression alone can explain reduced 5HT levels. MeCP2 was shown to greatly affect brain-derived neurotrophic factor (BDNF) (Li and Pozzo-Miller, 2014), which in turn is a neurotrophic factor for serotonergic neurons (Paterson et al., 2005). In this regard, restoring BDNF function in a mouse model of RTT had beneficial effects (Ogier et al., 2007). However, the up-regulation of *Htr1d* might also be a factor in the reduction of 5HT. 5-HT_1D_R was found as an auto-receptor on brainstem neurons, reducing their activity when activated by binding of serotonin. *Htr1d* up-regulation in the brainstem could therefore auto-inhibit the release of serotonin and thus contribute to low brain serotonin levels reported previously (Mokler et al., 1998).

We cannot exclude that the observed changes of *Ddc* and *Slc6a4* are mainly the result of a compensatory mechanism to counteract the dysregulation discussed above. An increase of dopa-decarboxylase (encoded by *Ddc*) should, although not the rate-limiting enzyme in 5HT synthesis (Kuhar et al., 1999; Berry, 2004) increase serotonin levels. Similarly, a serotonin transporter (5-HTT; *Slc6a4)* down-regulation will impair the re-uptake of serotonin from the synaptic cleft and this increases the extracellular levels. Nevertheless, a direct control of *Ddc expression* by MeCP2 is possible, as *Ddc* shows the same direction of dysregulation in brainstem and hippocampus.

Although *Ddc* is part of the serotonergic system, its contribution to the RTT phenotype must also be discussed with respect to its role in the dopaminergic system. Several groups reported decreased levels of dopamine in the brain (Jellinger, 2003) and dopamine in the basal ganglia is important for the execution of coordinated and smooth movement, which is disturbed in *Mecp2^-/y^* mice evidenced by hind limb clasping. As *Ddc* is also not the rate-limiting enzyme for dopamine synthesis, its upregulation here might again be compensatory.

In this regard, our results fit well with the established RTT pathology. The dysregulation of *Tph2* and *Htr1d* might directly contribute to low serotonin levels, while the dysregulation of *Slc6a4* and *Ddc* are, however, likely compensatory.

Taken together, our data points to the complex dysregulation of the serotonergic system at different levels, which can contribute to the functional dysregulation in the brain of MeCP2-deficient mice. This complexity is reflected by the fact that different strategies of pharmacotherapy seem to improve symptoms in RTT, although targeting opposing signaling pathways (reduction of cAMP via 5-HT_1A_; Abdala et al., 2010) or (elevation of cAMP via 5-HT_7_; De Filippis et al., 2014, 2015). Thus, it appears to be necessary to improve our knowledge of cell-type specific expression and regulation of serotonin-dependent GPCRs, not only in our mouse models but, of course, also in humans, to finally develop effective pharmacological tools.

## Author Contributions

SV and SN contributed equally. SV, SN, UR, WM, TM, and MN performed experiments. SN, SH, and MN wrote the manuscript. TM, SH, and MN conceived the study.

## Conflict of Interest Statement

The authors declare that the research was conducted in the absence of any commercial or financial relationships that could be construed as a potential conflict of interest.

## Footnotes

1 http://frodo.wi.mit.edu/primer3/
